# The impact of school-based creative bibliotherapy interventions on child and adolescent mental health: a systematic review and realist synthesis protocol

**DOI:** 10.1186/s13643-024-02482-8

**Published:** 2024-03-13

**Authors:** Hayley Redman, G. J. Melendez-Torres, Alison Bethel, Judith Green

**Affiliations:** 1 Wellcome Centre for Cultures and Environments of Health, University of Exeter, Exeter, UK; 2 Department of Public Health and Sport Sciences, Faculty of Health and Life Sciences, University of Exeter, Exeter, UK; 3 NIHR ARC South West Peninsula (PenARC) University of Exeter Medical School, Exeter, UK

**Keywords:** Bibliotherapy, Child and adolescent mental health, Schools

## Abstract

**Background:**

There is a need to identify evidence-based interventions to be delivered in schools that can be used to improve child and adolescent mental health and wellbeing. Creative bibliotherapy is one proposed intervention. However, there has been, to date, no comprehensive assessment of the evidence for its impact on mental health and wellbeing. To fill this gap, we will conduct a systematic review and realist synthesis.

**Methods:**

A systematic search of the bibliographic databases APA PsycINFO, Medline (via Ovid), CINAHL, ERIC, Education Research Complete (via EBSCOhost) and Web of Science (SCI, SSCI, AHCI, ESCI) for school-based creative bibliotherapy interventions on child and adolescent mental health. Types of study to be included: cohort studies, non-randomised comparative evaluations, randomised controlled trials. The data from all included studies will be summarised descriptively and strength of evidence appraised. This is a potentially large field of practice, with heterogeneous interventions; we will use methods from intervention components analysis to describe and categorise the range of components and approaches used in included interventions. To understand how interventions work and in which contexts, we will use methods from realist synthesis to develop an exploratory account of mechanisms in different settings and for different young people (contexts).

**Discussion:**

Findings will assess the range of evidence for the impact of creative bibliotherapy on child and adolescent mental health and wellbeing, the strength of evidence for the impact identified, and describe potential mechanisms. This review will be useful for a wide range of stakeholders considering implementing or developing interventions using creative bibliotherapy in school-based settings.

**Systematic review registration:**

This protocol was registered at the International Prospective Register of Systematic Reviews (https://www.crd.york.ac.uk/prospero/), registration number CRD42023410333. This review is funded by Wellcome Trust (221457/Z/20/Z).

**Supplementary Information:**

The online version contains supplementary material available at 10.1186/s13643-024-02482-8.

## Background

At least one in four of the UK population experience a mental health issue at some point in their lives [1]. *Future in Mind*, the report from the UK’s Children and Young People’s Mental Health and Wellbeing Taskforce, estimates that over half of mental health problems in adult life start by the age of 14 and 75% by age 18 ([1], p. 9). The report emphasises the need for evidence-based early intervention and multi-sectoral action. The report also stresses the importance of promoting mental health and wellbeing to everyone—not just focusing on mental illness and diagnosis. Universal services, such as schools, can play a key role in preventing mental health problems and promoting mental wellbeing. Furthermore, the effectiveness of taking a whole school approach to well-being has been shown for both physical and mental health and well-being outcomes, for example, body mass index, tobacco use, and being bullied ([1], p. 36).

To improve child and adolescent mental health and wellbeing, the UK Government has begun to implement strategies outlined in the 2019 NHS Long Term Plan [2] and the 2017 Green Paper on Transforming Children and Young People’s Mental Health Provision [3]. Several of these plans are relevant to promoting mental health and well-being in all children in schools, including the integration of mental well-being learning into the curriculum and ensuring a whole school approach to wellbeing. The National Institute for Health Care Excellence (NICE) guidance on *Social, emotional, and mental well-being in primary and secondary education* [4] recommendations include ensuring ‘that the curriculum for all pupils includes evidence-based, culturally appropriate information about social, emotional and mental wellbeing to develop children and young people’s knowledge and skills as part of the whole-school approach’ including the integration of ‘relevant activities into all aspects of education to reinforce the curriculum offer about social, emotional and mental wellbeing and skills’ and ‘universal interventions’ ([4], p. 9–10). Creative bibliotherapy could be an efficient and effective tool in a school’s arsenal to promote mental health and wellbeing. Suvilehto [5] argues that many teachers already practice bibliotherapy in some manner, without giving their practice a formal name. This systematic review will assess the range and quality of evidence for the effectiveness of creative bibliotherapy in school settings.

### Creative bibliotherapy

Bibliotherapy practice is a multifaceted and complex mixture of approaches and interventions operating under the broad banner of using books to heal ([6], p. 18). An analysis of the 100 most cited papers on bibliotherapy identified depression, anxiety, panic disorder, insomnia, and aphasia as key areas for bibliotherapy’s application [7].

There is no universally agreed definition of bibliotherapy–Hicks argues there is almost as much diversity in the definition of the term bibliotherapy as there is in its practice ([6], p. 13). Bibliotherapy spans a continuum,stretching from the use of creative literature to promote health and well-being at one end to clinical intervention and psychological therapy at the other, with considerable variation in between ([6], p. 13). Sitting on either end of this continuum, Brewster [8] presents two distinct models of bibliotherapy: *self-help* and *creative,* representing a synthesis of models from the literature that also reflect current practice in the UK. These terms have since been widely used in the literature (for example [9, 10]):

- *Self-help* bibliotherapy: the use of nonfiction self-help books, often recommended by medical practitioners, to provide practical help to people with mental health problems.
- *Creative* bibliotherapy: the use of fiction and poetry to work with individuals and groups to promote better mental health.

Creative bibliotherapy can be delivered in a number of ways. There are two key models of creative bibliotherapy currently practiced in settings such as the UK. The first stresses the individual and individual reading—the right book must be found for the right person at the right time. This model is practiced by The Reading Agency, a UK-based charitable organisation that works with partners in both the health and education sectors to promote using the ‘proven power of reading’ to help people tackle ‘life’s big challenges’ [11]. The second concentrates on reading literary works from *the canon,* that is, classic texts in English, such as the works of Jane Austen, Charles Dickens, and William Shakespeare, aloud in a group setting to facilitate discussion. This model is practiced by The Reader, another UK-based charity that works to bring people together, including schools and families, to ‘experience and enjoy great literature, which [they] believe is a tool for helping humans survive and live well’ [12]. Troscianko and colleagues highlight an interesting divergence in the literature: whilst the majority of existing bibliotherapy theory entails individual reading, most empirical work has assessed group reading [13].

This systematic review will focus on *creative* bibliotherapy. C*reative* bibliotherapy is defined as: the reading and discussion of creative texts including, but not limited to, fiction books, short stories, picture books, and poetry, including alternative formats (e.g. e-books). In comparison to the literature on self-help bibliotherapy, the evidence base for creative bibliotherapy is much smaller and more eclectic. Although the practice has been dated back to ancient Greek medicine [14], the UK National Association of Primary Care (NAPC) notes the word was rarely used in medical practice until 2004, when the UK National Institute for Health and Care Excellence (NICE) produced new guidelines for depression [15]. These guidelines suggested that for patients with mild depression, healthcare professionals should consider recommending a guided self-help programme based on cognitive behavioural therapy (CBT). Hicks [6] notes that this was driven in part by the UK’s socio-political context of ‘a health sector charged with becoming more productive, using resources more effectively, building capacity and engaging people in taking responsibility for their own health’ in the early 2000s ([6], p. 13).

There is an established evidence base for the effectiveness of self-help bibliotherapy for a range of mental health and wellbeing outcomes at all ages (e.g. [16, 17]). However, the evidence base for creative bibliotherapy is far less well-developed. Furthermore, Troscianko [9] has argued that current research and practice of creative bibliotherapy is underdeveloped in its understanding of the mechanisms of change. Existing theories are based on minimal empirical evidence and are largely based on the individual reader paradigm [13].

Preliminary searches located two systematic reviews that have examined evidence of the impact of creative bibliotherapy on mental health and wellbeing. The first, from Montgomery and Maunders, investigates the effectiveness of creative bibliotherapy for internalising, externalising, and pro-social behaviours in children [10]; the second, from Glavin and Montgomery, reviewed creative bibliotherapy for post-traumatic stress disorder [18]. The eight randomised controlled trials included in Montgomery and Maunders’ review suggest creative bibliotherapy has a small to moderate positive effect on child behaviour [10]. No studies met the inclusion criteria for the second review. The authors note that whilst excluded studies (*N* = 13) provided valuable qualitative information regarding bibliotherapy’s acceptability and utility, they lacked a robust study design [18]. Neither review aimed to describe the mechanisms of change. However, Montgomery and Maunders note that ‘Although no definitive model of creative bibliotherapy emerges from the included studies, all interventions reflected to some extent the evidence-based steps of CBT’ [10]. They suggest that each creative text used ‘provided opportunity for identification of unhelpful beliefs and behaviours, challenging of their meaning, and the development of new beliefs and behaviours’ [10]. In the later systematic review, Glavin and Montgomery propose that this transporting effect of literary reading may also explain a ‘possible causal linkage between reading and PTSD [post-traumatic stress disorder] treatment through the lens of prolonged exposure techniques’ in which phenomena that would be threatening in the real world can be safely engaged within the fictional one [18]. These theories are discussed below.

### Mechanisms of change

The realist synthesis will develop a provisional programme theory for creative bibliotherapy, drawing on evidence from the studies included in the systematic review, and also on the existing literature on mechanisms of change. For *self-help* bibliotherapy, Troscianko [9] argues there is an underlying assumption that (if) it works, it works because the therapeutic model (cognitive behavioural therapy [CBT]) it is based on works. Some suggest similar mechanisms for creative bibliotherapy. Dwivedi and Gardner argue that experiencing stories through fiction, poetry, and film could act on these same CBT mechanisms, teaching ‘new attitudes and belief systems’ [19]. During reading, both cognitive processes and emotional processes occur [20, 21]. Cognitive processes such as recognition and reframing are key to the recognition of unhelpful cognitions and, as such, elicit more realistic thoughts and assumptions. Emotional processes, such as empathy and identification, allow for previously unconsidered and unhelpful cognitions to surface, allowing the reader to be challenged with new ways of interpreting these through insight into a fictional world. The reader begins ‘to understand others and their plights from perspectives other than [their] own’ ([20], p. 62).

Creative texts can emotionally transport the reader into a story that is both pleasurable and rewarding, with certain stories providing an opportunity to engage safely with emotional difficulties while the characters the reader connects with deal with their own [20]. Empirical research has shown that fiction is processed differently from non-fiction, with a respective difference in brain activation; for more on neural mechanisms of change see, e.g. Tribe et al. [22]. It is proposed that fiction improves our ability to understand other people’s perspectives, due to the way our brains process and comprehend narratives [23]. This is theorised as the ‘transportation effect’ whereby stories have the power to transport readers from the real to the narrative world [24]. Green argues that transportation into the narrative world ‘can lead to real-world belief (and behaviour) change’ [24]. Similarities, both demographic and between the reader’s life and the character’s story, may lead to a stronger sense of transportation ([25], p. 27). Sharing the experience with others in the real world (as in the group bibliotherapy model outlined above) allows the reader to form connections and community, considered building blocks in mental health recovery [26].

**Fig. 1:**
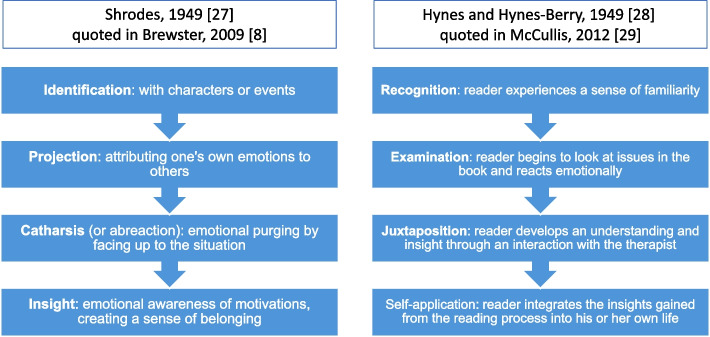
Models of bibliotherapy [27, 28]

Shrodes [27] and Hynes and Hynes-Berry [28] present models of the conceptual effects and processes involved in successful bibliotherapy [8, 29]. These models are presented in Fig. 1.

Both of these models are based on the individual reader paradigm, which Troscianko and colleagues [13] argue suggest as emphasising the similarity between the reader, their ‘problem’ and the arc of the protagonist’s story. This similarity prompts a connection between the reader and the protagonist, provoking the subsequent stages of these models. Troscianko and colleagues critique the shortcomings of these models based on the individual reader paradigm, which faces both ‘empirical and theoretical obstacles’ for explaining ‘how and to what extent ‘similarity’ is therapeutically beneficial’ [13].

Jones [30] proposed core processes across the creative art therapies (CATs) including artistic projection, perspective and distance, embodiment, non-verbal experience as detailed by others, but also the playful space and the informed player, the participating art therapist, the active witness, and the triangular relationship. This theory places significant weight on the participant-therapist relationship and interaction. Research from the *self-help* model has tested the efficacy of various degrees of therapist involvement in bibliotherapy. This is often in the context of drivers to find low-cost alternatives to traditional therapy or to reduce contact hours. A meta-analysis of self-help interventions in the management of depressive symptoms, however, found ‘pure’ (as opposed to ‘guided’ self-help) had a minimal effect size (0.06) on depression [31]. There are concerns that this may have led to an exaggeration of the effectiveness of bibliotherapy without facilitation [32]. There is little research on the extent to which therapeutic contact drives the effectiveness of creative bibliotherapy. Furthermore, although noted by Billington et al. [33] as an essential ‘mechanism of action’, the extent to which the group setting drives change has not come under scrutiny in the literature. Any impact of these interactions (either with a therapist or within a group) could be attributed to Lazarus and Folkman’s ‘transactional model of stress and coping’ which contends a person’s capacity to cope and adjust to challenges and problems is a consequence of transactions (or interactions) that occur between a person and their environment [34].

Across the literature, for both self-help and creative bibliotherapy, little attention is paid to the actual content of the literature used for bibliotherapy. Brewster notes in most studies the only information provided about the text is ‘the number of pages in the book and a quantified reading age’ making it difficult to assess the importance of linguistic style and for self-help texts the ‘therapeutic approach and balance of instruction and reflection that facilitates effective treatment’ ([35], p. 9–10). Other than brief explanations of what the canon includes and a presumption that these texts contain narratives to which all can relate, there is a lack of information available on what books are used for creative bibliotherapy in studies. However, the individual reader paradigm would argue against such a universalised approach. Research by McNicol [36] concerning the reception of Arthur Frank’s illness narratives, found that restitution and chaos narratives were not well received: they were deemed unsatisfying, unsettling, and unhelpful. Narratives describing how to accept, live with and better understand health conditions were much better received, described by Frank as stories that ‘tell of searching for alternative ways of being ill’ ([37], p. 117). Again, this assumes an ‘identification’ based connection between the reader and protagonist which we neither know how or to what extent this is therapeutically beneficial [13].

Furthermore, a consideration of the book as a symbol or material object is not covered in the existing literature. Does the act of receiving a physical book specifically chosen for you or a personalised recommendation offer on some level the feeling of being cared for? If a patient was prescribed a book by a GP would this feel more personal and caring than a ‘regular’ prescription of pills? Although unconfirmed by the data generated, Lundmark hypothesised the Bible, for example, serves two functions as a coping tool: a collection of stories that bear meaning to the reader; and as an artifact, with the focus on the Bible as a ‘tangible physical object’ ([38], p. 142). This suggests there is scope for testing the significance of the book as a material object to the outcome of bibliotherapy interventions. As such, we will include interventions that use both physical books and alternative formats (e.g. e-books), and the realist synthesis will allow consideration of the effects of the book as material object.

Troscianko and colleagues highlight how the theoretical models from the individual reading paradigm tend to follow a common pattern: they emphasise the similarity between the reader’s problematic experience and the arc of the protagonist’s story [13]. Billington et al. [33], propose a set of four significant ‘mechanisms of action’ based on an evaluative study of *group* creative bibliotherapy. The first three were deemed essential to its success, while the fourth was considered influential: (1) ‘A rich, varied, non-prescriptive diet of serious literature’ including a mix of fiction and poetry. Both literary forms allowed participants to discover (or rediscover) modes of thought, feeling, and experience. (2) The group facilitator’s role ‘in expert choice of literature, in making the literature ‘live’ in the room and become accessible to participants through skillful reading aloud, and in sensitively eliciting and guiding the discussion of the literature’. The facilitator’s alert presence in relation to literature, the individual and the dynamics of the group is a complex and crucial element of the intervention. (3) The group’s role ‘in offering support and a sense of community’, discussions elicited in response to the texts allowed personal ideas, feelings, opinions and experiences to be shared, which was ‘demonstrably critical in ‘knitting’ the group together’. (4) The environment in contributing to the ‘atmosphere, group dynamic and expectation of the utility of the reading group’. This study identified that a group located in a mental health drop-in centre were ‘much more willing to engage with the literature for its own sake from the very outset of the study’ in comparison to a group located in a GP surgery, who tended to view the literature as something ‘prescribed’ [33]. These four mechanisms, however, are challenging to evaluate, as it is difficult to determine if the observed effects are due to all, none, or any subset of these four factors. Furthermore, the importance of the four factors in different configurations may be highly variable in different contexts.

By drawing on realist thinking of causation, we will identify indicative causal processes (i.e. mechanisms) that lead to the impacts of bibliotherapy. Where possible we will compare the causal processes we identify to those theorised in the literature, and by doing so potentially refine our understanding of bibliotherapy.

## Methods/design

This protocol was registered at the International Prospective Register of Systematic Reviews (https://www.crd.york.ac.uk/prospero/), registration number CRD42023410333. The protocol is being reported in accordance with the Preferred Reporting Items for Systematic Review and Meta-Analysis Protocols (PRISMA-P) statement, attached in Additional file 1.

### Objectives

The systematic review and realist synthesis will answer the following questions:

- What are the impacts of school-based creative bibliotherapy interventions on child and adolescent mental health?
- What mechanisms can be identified through which impacts are achieved, and in which contexts?

### Eligibility criteria

#### (P) Population

5–16-year-old children and adolescents attending mainstream schools—the equivalent to primary and secondary school ages in the UK.

#### (I) Intervention

School-based interventions that use creative bibliotherapy to improve children and adolescents’ mental health and wellbeing. Creative bibliotherapy involves the reading and discussion of creative texts including, but not limited to, fiction books, short stories, picture books, and poetry, including alternative formats (e.g. e-books). Non-fiction, didactic and self-help texts will be excluded.

Interventions must include literature-related components, as defined above. No restrictions will be placed on the discussion format (facilitated group discussion, peer-to-peer, individual with teacher/librarian) or setting within the school (class-based, whole school, small group). Interventions will be excluded if they are designed to be delivered by a clinical practitioner/health professional.

#### (C) Comparator

Studies will be included that include any valid comparator e.g. other intervention, a waitlist control, do nothing, or treatment as usual.

#### (O) Outcome

All mental health and wellbeing outcomes will be included, including, but not limited to depressive symptomatology, anxious symptomatology, internalising problems, externalising problems, conduct disorders, disruptive behaviour. Additional outcomes will include mediators of mental health and mental wellbeing, including but not limited to self-concept, self-efficacy, and mindfulness.

### Information sources

The following bibliographic databases will be searched: APA PsycINFO, Medline (via Ovid), CINAHL, ERIC, Education Research Complete (via EBSCOhost) and Web of Science (SCI, SSCI, AHCI, ESCI).

### Search strategy

The searches will include both free text terms and controlled vocabulary terms when available and appropriate, these will be developed by the information specialist. No date or language restrictions will be applied at the searching stage. Forwards and backward citation searching will also be undertaken in Scopus using the final included articles from the database searches. A draft search strategy is available in Additional file 2.

### Study records

#### Data management

Details of all searches will be recorded. Search results will be downloaded to EndNote desktop software. Studies sourced through supplemental hand searching will be recorded and imported into EndNote.

#### Selection and data collection process

In the first screening, two reviewers will independently screen titles, abstracts and keywords of all the studies yielded by the search against the inclusion/exclusion criteria, displayed in Table 1, using Rayyan [39].

**Table 1:** Inclusion and exclusion criteria

**Inclusion criteria**	**Exclusion** criteria
• Interventions that use creative texts (fiction books, stories, poetry) • Interventions based in mainstream schools • Interventions designed to be delivered by non-clinical practitioners (e.g. teacher, librarian, pastoral support worker) • Mental health and wellbeing outcomes • Non-randomised comparative evaluations, randomised controlled trials, interrupted time series	• Interventions that use non-fiction, didactic, or self-help texts • Interventions designed to be delivered by clinical practitioners and/or healthcare professionals • Interventions based in specialist schools or delivered outside the school • Interventions that do not include the discussion of texts • Case studies, opinion papers, conference papers, and any other research without primary data.

Studies that fail to meet the inclusion criteria and any duplicates will be excluded, and full texts will be obtained for studies that appear to meet the inclusion criteria or for which there is any uncertainty. Full texts will then be screened independently by two reviewers to determine whether they meet the inclusion criteria, with reasons for exclusion noted. Disagreements will be resolved by a third reviewer.

Data for the systematic review will be extracted from included studies to map the range of evidence, assess quality in terms of risk of bias, and identify evidence for a realist synthesis. The latter will include evidence relating to a provisional programme theory for creative bibliotherapy. Data extraction will be performed by one reviewer using a data extraction form developed by the researchers for the purposes of this review. This form will be refined by the reviewer until the data extraction is complete, to ensure the appropriateness and usefulness of all fields.

#### Risk of bias in individual studies

Quality appraisal for the systematic review will be carried out by two members of the review team using the Cochrane tools for risk of bias in randomised and non-randomised studies. However, as this systematic review is aiming to map the range of evidence for school-based creative bibliotherapy no studies will be excluded on the grounds of quality.

#### Data synthesis: mapping the evidence

The data from all included studies will be summarised descriptively. Tables and text will provide key study characteristics which will be summarised and appraised. Details will include study characteristics (first author, publication year, origin), study design (sample size, population characteristics, risk of bias assessment criteria), intervention features (duration, mode of delivery), drop-out rates and assessment tools for primary and secondary outcomes.

Because these interventions are heterogeneous, we will use methods from intervention components analysis [40] to describe and categorise the range of components and approaches used in the included intervention.

#### Data synthesis: identifying potential mechanisms and contexts

Subsequently, to understand how interventions work and in which contexts, we will undertake a realist synthesis [41] to develop an exploratory account of how these interventions work (mechanisms) in different settings and for different young people (contexts). Capacity precludes a full realist synthesis: this will be restricted to the data from studies included in the systematic review, and theoretical evidence on potential mechanisms of change from selected theoretical studies (see Mechanisms of Change, above). We will develop this account using methods of constant comparison, working in pairs to consider the included evidence, and relate these to different intervention strategies identified in the components analysis. Reviewers will seek out the contextual (C) influences that are hypothesised to have triggered the relevant mechanism(s) (M) to generate the outcome(s) (O) of interest [41]. Synthesis will consist of developing an initial programme theory, informed by our narrative review of literature and an iterative process of refining this from explicit accounts from studies in the systematic review. We will then compare ‘how the programme was supposed to operate’ to the ‘empirical evidence on the actuality in different situations’—all along C-M-O lines. Analytic purchase comes from the ability to describe and understand the many contingencies that affect the likelihood of such interventions generating their intended outcomes [41]. In turn, this will provide exploratory indications about what schools might need to put into place to ensure the intervention is most likely to trigger the right mechanism(s) to produce the desired outcomes. This will contribute to a refined programme theory, which can be tested in future evaluations.

## Discussion

This protocol describes a planned systematic review of comparative studies of creative bibliotherapy interventions to understand the impact of school-based creative bibliotherapy interventions on child and adolescent mental health, with a realist synthesis. We are aware that this draws on two rather different paradigms of evaluation. Whereas the comparative studies included in the systematic review draw on probabilistic methods for identifying effect sizes, a full realist review would draw on a wider range of evidence, selected for its capacity to address and then refine the programme theory [42], and underpinned with a generative model of causality. There are good epistemological grounds for rejecting claims of ‘realist’ perspectives in reviews in which the underlying evidence is derived from probabilistic causal designs [43]. However, we believe that our synthesis can draw on some of the insights of a more realist perspective, using a narrower set of evidence. This combination has been used effectively in studies of similar topics, such as therapeutic writing [44]. Our synthesis will generate indicative insights on the mechanisms and contexts that are important to consider when designing future interventions of creative bibliotherapy. By undertaking a realist synthesis, even of a somewhat selective body of evidence, this review will provide an initial exploratory account of how these interventions work in different settings and for different people—the context-mechanism-outcome (CMO) configurations—to help us understand how, why, and for whom an intervention produced the desired and undesired outcomes. Capacity prevents a full realist review, and we recognise that by restricting the documents eligible for inclusion, we will be providing exploratory causal explanations. These can be used in future empirical studies to confirm, refute, or refine theorised CMOs. When completed, the findings of the systematic review may be of interest to educational professionals, health and social care practitioners, commissioners and providers, as well as professionals who work in the voluntary and community sectors. These will also provide an initial programme theory for testing in future evaluations of creative bibliotherapy.

## Strengths

The systematic review will identify evidence of impact from studies with a counterfactual. A realist synthesis will shed light on the mechanisms of change for creative bibliotherapy, which is currently underdeveloped in the literature.

## Limitations

Given the lack of clarity in distinguishing between creative and self-help bibliotherapy in the literature, it can be difficult to deduce which bibliotherapy is being referred to in some texts.

Inclusion criteria restricted to studies with a comparator will potentially exclude more holistic appraisals of creative bibliotherapy. Capacity precludes a full realist synthesis, which might furnish better developed theory about the interaction of context and mechanisms.

### Supplementary Information

**Supplementary Material 1.**

**Supplementary Material 2.**

## Data Availability

Data sharing is not applicable to this article as no datasets were generated or analysed during the current study.

## References

[1] Department of Health. Future in mind: promoting, protecting and improving our children and young people’s mental health and wellbeing. London: Department of Health; 2015. Available at: https://assets.publishing.service.gov.uk/government/uploads/system/uploads/attachment_data/file/414024/Childrens_Mental_Health.pdf.

[2] NHS. The NHS long term plan. London: NHS; 2019. Available at: https://www.longtermplan.nhs.uk/publication/nhs-long-term-plan/.

[3] Greening J, Hunt J. Transforming children and young people’s mental health provision: a green paper. London: Department of Health and Social Care and Department for Education; 2017. Available at https://www.gov.uk/government/consultations/transforming-children-and-young-peoplesmental-health-provision-a-green-paper.

[4] NICE. Social, emotional and mental wellbeing in primary and secondary education*.* London: National Institute for Health and Care Excellence. Available at: https://www.nice.org.uk/guidance/ng223. 36787393

[5] Suvilehto P (2019). We need stories and bibliotherapy offers one solution to developmental issues. Online J Complement Altern Med.

[6] Hicks D (2006). An audit of bibliotherapy/books on prescription activity in England.

[7] Xu Z, Liu R, Guo L, Gao Z, Gao Z, Liu X, Li J, Li B, Yang K. The 100 most-cited articles on bibliotherapy: a bibliometric analysis. Psychol Health Med. 2022;28(9):1–7. 10.1080/13548506.2022.2068183 35473482

[8] Brewster L (2009). Books on prescription: bibliotherapy in the United Kingdom. J Hosp Librariansh.

[9] Troscianko ET (2018). Fiction-reading for good or ill: eating disorders, interpretation and the case for creative bibliotherapy research. Med Humanit.

[10] Montgomery P, Maunders K (2015). The effectiveness of creative bibliotherapy for internalizing, externalizing, and prosocial behaviors in children: a systematic review. Child Youth Serv Rev.

[11] Readingagency.org. UK: the reading agency: about. 2023. Available at: https://readingagency.org.uk/about/. [cited 20 Apr 2023].

[12] Thereader.org. UK: The reader: what we do. 2023. Available at: https://www.thereader.org.uk/what-we-do/. [cited 20 Apr 2023].

[13] Troscianko ET, Holman E, Carney J (2022). Quantitative methods for group bibliotherapy research: a pilot study. Wellcome Open Res.

[14] Brewster L (2008). Medicine for the soul: bibliotherapy. Austral Public Libr Inform Serv.

[15] NAPC. Reading Well: books on prescription: how bibliotherapy can help your patients and save your practice time and money. London: National Association of Primary Care; 2018. Available at: https://napc.co.uk/wp-content/uploads/2017/09/Reading-well.pdf.

[16] Moldovan R, Cobeanu O, David D (2013). Cognitive bibliotherapy for mild depressive symptomatology: randomized clinical trial of efficacy and mechanisms of change. Clin Psychol Psychother.

[17] Lewis KM, Amatya K, Coffman MF, Ollendick TH (2015). Treating nighttime fears in young children with bibliotherapy: evaluating anxiety symptoms and monitoring behavior change. J Anxiety Disord.

[18] Glavin CE, Montgomery P (2017). Creative bibliotherapy for post-traumatic stress disorder (PTSD): a systematic review. J Poet Ther.

[19] Dwivedi K, Gardner D (1997). ‘Theoretical perspectives and clinical approaches’ in Dwivedi, K. The Therapeutic Use of Stories.

[20] Oatley K (1995). A taxonomy of the emotions of literary response and a theory of identification in fictional narrative. Poetics.

[21] Oatley K (1999). Meetings of minds: dialogue, sympathy, and identification, in reading fiction. Poetics.

[22] Tribe KV, Papps FA, Calvert F (2021). “It just gives people hope”: a qualitative inquiry into the lived experience of the Harry Potter world in mental health recovery. Arts Psychother.

[23] Altmann U, Bohrn IC, Lubrich O, Menninghaus W, Jacobs AM (2014). Fact vs fiction—how paratextual information shapes our reading processes. Soc Cogn Affect Neurosci.

[24] Green MC (2006). Narratives and cancer communication. J Commun.

[25] McNicol S (2018). ‘Theories of bibliotherapy’ in Brewster L, McNicol S. Bibliotherapy.

[26] Leamy M, Bird V, Le Boutillier C, Williams J, Slade M (2011). Conceptual framework for personal recovery in mental health: systematic review and narrative synthesis. Br J Psychiatry.

[27] Shrodes C (1949). Bibliotherapy: a theoretical and clinical-experimental study.

[28] Hynes A, Hynes-Berry M (1986). Bibliotherapy: the interactive process a handbook.

[29] McCulliss D (2012). Bibliotherapy: historical and research perspectives. J Poet Ther.

[30] Jones P. The arts therapies: a revolution in healthcare. Abingdon, New York: Routledge; 2020.

[31] Gellatly J, Bower P, Hennessy SU, Richards D, Gilbody S, Lovell K (2007). What makes self-help interventions effective in the management of depressive symptoms? Meta-analysis and meta-regression. Psychol Med.

[32] Febbraro GA (2005). An investigation into the effectiveness of bibliotherapy and minimal contact interventions in the treatment of panic attacks. J Clin Psychol.

[33] Billington J, Dowrick C, Hamer A, Robinson J, Williams C (2010). An investigation into the therapeutic benefits of reading in relation to depression and well-being.

[34] Lazarus RS, Folkman S. Stress, appraisal, and coping. New York; Springer Publishing Company; 1984.

[35] Brewster L (2018). ‘Bibliotherapy: a critical history’ Brewster L, McNicol S. Bibliotherapy.

[36] McNicol S. The impact of educational comics on feelings and attitudes towards health conditions. Manchester Metropolitan University [9 July 2020]. 2015.

[37] Frank AW. The wounded storyteller: body, illness, and ethics. Chicago: University of Chicago Press; 2013.

[38] Lundmark M (2019). The Bible as coping tool: Its use and psychological functions in a sample of practicing Christians living with cancer. Arch Psychol Relig.

[39] Ouzzani M, Hammady H, Fedorowicz Z, Elmagarmid A (2016). Rayyan—a web and mobile app for systematic reviews. Syst Rev.

[40] Sutcliffe K, Thomas J, Stokes G, Hinds K, Bangpan M (2015). Intervention Component Analysis (ICA): a pragmatic approach for identifying the critical features of complex interventions. Syst Rev.

[41] Wong G, Westhorp G, Pawson R, Greenhalgh T (2013). Realist synthesis. RAMESES training materials.

[42] Pawson R, Greenhalgh T, Harvey G, Walshe K (2004). Realist synthesis-an introduction. ESRC Res Methods Prog.

[43] Marchal B, Westhorp G, Wong G, Van Belle S, Greenhalgh T, Kegels G, Pawson R (2013). Realist RCTs of complex interventions–an oxymoron. Soc Sci Med.

[44] Nyssen OP, Taylor SJ, Wong G, Steed E, Bourke L, Lord J, Ross CA, Hayman S, Field V, Higgins A, Greenhalgh T (2016). Does therapeutic writing help people with long-term conditions? Systematic review, realist synthesis and economic considerations. Health Technol Assess.

