# Supplementary material for: The impact of school-based creative bibliotherapy interventions on child and adolescent mental health: a systematic review and realist synthesis protocol
Source: Syst Rev. 2024 Mar 13;13:86. doi: 10.1186/s13643-024-02482-8 (PMC10936023; doi:10.1186/s13643-024-02482-8)
Supplement: Supplementary file 2 — Supplementary Material 2. [file 13643_2024_2482_MOESM2_ESM.pdf]

Example Search Strategy

The impact of school based creative bibliotherapy interventions on child and adolescent mental health: a systematic review and realist synthesis

Search strategy for APA PsycINFO

1. school*.ti,ab. (429373)
2. education*.tw. (555392)
3. academ*.tw. (193086)
4. (classroom* or class or classes).tw. (244565)
5. grade*.tw. (167532)
6. lesson*.tw. (41423)
7. (primary or secondary).tw. (351967)
8. adolescent/ or exp child/ (0)
9. Adolescen*.tw. (292268)
10. teen*.tw. (25567)
11. student*.tw. (655100)
12. pupil*.tw. (28794)
13. youth*.tw. (120969)
14. youngster*.tw. (3842)
15. (boy or boys).tw. (79303)
16. girl*.tw. (81584)
17. child*.tw. (789779)
18. (young* adj2 (adult* or person* or individual* or people* or population* or man or mans or men or mens or woman* or women*)).tw. (131403)
19. 8 or 9 or 10 or 11 or 12 or 13 or 14 or 15 or 16 or 17 or 18 (1621752)
20. bibliotherap*.tw. (1342)
21. (((story or stories) adj5 (book or books or fiction* or creativ*)) or storybook*).tw. (4394)
22. (reading adj4 (book or books or fiction* of character* or recreation* or poet* or poem*)).tw. (7653)
23. (listen* adj4 (stor* or book or books or fiction* or poet* or poem*)).tw. (1712)
24. Bibliotherapy/ (818)
25. 20 or 21 or 22 or 23 or 24 (14544)
26. 2 or 3 or 5 or 6 or 7 (1080296)
27. 19 and 26 (559076)
28. 1 or 4 (596545)
29. 27 or 28 (887367)
30. exp Mental Health/ (86883)
31. (mental* or psychiatric or emotion* or stress* or distress* or psychologic* or phobic* or compuls* or depress* or mood* or panic* or trauma* or anxi* or fear* or wellbeing or well being).tw. (1841183)
32. 30 or 31 (1842093)
33. 25 and 29 and 32 (1213)
